# Temperature and humidity associated with increases in tuberculosis notifications: a time-series study in Hong Kong

**DOI:** 10.1017/S0950268820003040

**Published:** 2020-12-28

**Authors:** M. Xu, Y. Li, B. Liu, R. Chen, L. Sheng, S. Yan, H. Chen, J. Hou, L. Yuan, L. Ke, M. Fan, P. Hu

**Affiliations:** 1 Key Laboratory of Environment and Health (HUST), Ministry of Education & Ministry of Environmental Protection, and State Key Laboratory of Environmental Health (Incubation), School of Public Health, Tongji Medical College, Huazhong University of Science and Technology, Wuhan 430030, Hubei, China; 2 School of Nursing, Hubei University of Chinese Medicine, Wuhan 430030, Hubei, China; 3 Department of Tuberculosis Prevention, Wuhan Pulmonary Hospital, Wuhan 430030, Hubei, China; 4 Centre of Health Administration and Development Studies, Hubei University of Medicine, Shiyan 442000, Hubei, China; 5 Faculty of Education, Health and Wellbeing, University of Wolverhampton, Wolverhampton, UK; 6 Department of Stomatology, Affiliated People's Hospital of Hubei Medical College, Shiyan 442000, Hubei, China; 7 Department of Epidemiology and Biostatistics and State Key Laboratory of Environment Health, Huazhong University of Science and Technology, Wuhan 430030, Hubei, China; 8 Biological Products Management Office, Hubei Provincial Centre for Disease Control and Prevention, Wuhan, Hubei, China; 9 Department of Epidemiology and Biostatistics, College of Public Health, Zhengzhou University, Zhengzhou 450052, Henan, China; 10 Shanghai University of Medicine & Health Sciences, Affiliated Sixth People's Hospital South Campus, Shanghai 201499, China

**Keywords:** Relative humidity, temperature, tuberculosis notification

## Abstract

Previous studies have revealed associations of meteorological factors with tuberculosis (TB) cases. However, few studies have examined their lag effects on TB cases. This study was aimed to analyse nonlinear lag effects of meteorological factors on the number of TB notifications in Hong Kong. Using a 22-year consecutive surveillance data in Hong Kong, we examined the association of monthly average temperature and relative humidity with temporal dynamics of the monthly number of TB notifications using a distributed lag nonlinear models combined with a Poisson regression. The relative risks (RRs) of TB notifications were >1.15 as monthly average temperatures were between 16.3 and 17.3 °C at lagged 13–15 months, reaching the peak risk of 1.18 (95% confidence interval (CI) 1.02–1.35) when it was 16.8 °C at lagged 14 months. The RRs of TB notifications were >1.05 as relative humidities of 60.0–63.6% at lagged 9–11 months expanded to 68.0–71.0% at lagged 12–17 months, reaching the highest risk of 1.06 (95% CI 1.01–1.11) when it was 69.0% at lagged 13 months. The nonlinear and delayed effects of average temperature and relative humidity on TB epidemic were identified, which may provide a practical reference for improving the TB warning system.

## Introduction

Tuberculosis (TB) is caused by *Mycobacterium tuberculosis* and has been documented as the greatest killer caused by a single-infectious pathogen worldwide in the past 5 years [1]. TB is influenced by a series of environmental and host-related risk factors, such as poor socioeconomic circumstances, overcrowding, smoking, the human immunodeficiency virus pandemic, diabetes mellitus, and other conditions that weaken the host immune system. TB incidence also varies by seasonal and spatial geographic factors [2, 3], implying that meteorological factors shape its seasonal and regional differences probably by affecting the transmission process of *M. tuberculosis*.

Recently, associations of meteorological factors with TB cases have been reported in some ecological studies, including temperature, relative humidity, sunshine duration, rainfall and wind speed [4–7]. For example, a study in Brazilian Federal District revealed that TB was more prevalent with temperatures of 20–23 °C, relative humidity of 31–69%, and ultraviolet radiation over 17 megajoules per square meter (MJ/m^2^) [4]. A spatiotemporal study of mainland China showed that rainfall, maximum wind speed, and sunshine duration were positively correlated with the total number of TB cases [5]. However, climate factors have obvious delayed impacts on TB incidence, but limited studies have taken their delayed impacts into consideration. For instance, a study in Birmingham found that a decrease in sunshine duration in winter was associated with the peak of TB incidence 6 months later [6]. Xiao *et al*. found that total rainfall and minimum relative humidity were inversely associated with TB incidence at lags of 4 and 3 months, respectively [7].

The distributed lag non-linear model (DLNM) can model the association of predictors with outcomes as well as the lag effects of predictors [8], and has recently been used to study the associations between meteorological factors and infectious diseases. But only two recent studies examined lag effects of climate factors on the TB cases based on this model. Xiao *et al*. estimated effects of climate factors on TB incidence in a Chinese southwestern city using monthly data, but their model was on the basis of a max lag of only 4 months [7]. Kuddus *et al*. analysed the association between climate factors and TB cases in North-East Bangladesh based on quarterly time-series [9]. Given that the median incubation period for TB was 15.33 months [10], the median delay from initial symptoms to initial treatment for TB was 49 days [11] and the existing gaps in previous studies mentioned above, we conducted a time-series study using a DLNM with a max lag of 20 months to explore the potential exposure–response between meteorological factors and the number of TB notifications, as well as their lag effects, in Hong Kong.

## Methods

### Study area

Hong Kong (centre: 114°15′E, 22°15′N), an international metropolis, which is located on the coast of South China, is among the world's most densely populated regions. It is an international financial, shipping and trade centre, and has extensive external exchanges in tourism and business, especially with mainland China. This city has a four-season oceanic subtropical monsoon climate. It is made up of Hong Kong Island, Kowloon, the New Territories, and surrounding 262 islands, covering 1106.34 km^2^ with permanent residents of 7 482 500 million in 2018.

### Environmental data collection

The monthly averages in meteorological indicators (including average temperature and relative humidity) of the whole city from 1997 to 2018 were derived from the Hong Kong Observatory [12]. The positions of 26 weather stations that had monitored both average temperature and relative humidity in Hong Kong during the study periods were illustrated in the map (Supplementary Fig. S1).

The monthly air pollution concentrations from 1997 to 2018 in each air quality monitoring station were obtained from the Hong Kong Environment Protection Department, and were averaged across all the 16 monitoring stations to get monthly averages of air pollution concentrations of the whole city, including PM_10_, PM_2.5_, NO_2_, SO_2_ and CO [13].

### Data on TB

The monthly TB notification statistics in Hong Kong from 1997 to 2018 were obtained from the Centre for Health Protection, Department of Health in Hong Kong, who kept the data in the TB notification registry system [14]. Diagnosis of TB mainly included radiological examination, microbiological tests, histological diagnosis, tests for TB-related biochemical and immunological markers, tests for TB infection (such as tuberculin skin test and T-cell-based gamma-interferon assays) and empirical trial of anti-TB drugs. Although limited geographic and demographic information for TB patients was obtained, the percent of permanent residents was >73% among the TB patients as indicated in the data from the Tuberculosis and Chest Service, Public Health Service Branch [15]. Therefore, for ecological studies, the meteorological and pollutant data we obtained can be considered to largely represent the exposure levels of TB patients notified during the study period. The population data for this area during the study period were retrieved from the Census and Statistics Department, Government of the Hong Kong Special Administrative Region. As this study used aggregate counts of TB cases notified, which involved no identical information of participants, no ethical approval was required.

### Statistical method

The association between meteorological factors and the number of TB notifications was modelled using a quasi-Poisson regression that allowed for overdispersion with DLNM [8]. Suppose *Y_t_* refers the monthly counts of TB notifications at calendar time (month) *t* ∈ (1, 2, …, 12) in Hong Kong and follows the Poisson distribution of *Y_t_*|*μ_t_* ~ POI(*μ_t_*), where *μ_t_* is the expected value of *Y_t_*. The model for evaluating the relationship between average temperature, relative humidity and the number of TB cases notified is described as follows:1
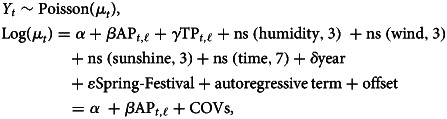
where *α* is the intercept; the two cross-basis functions *f*(TP, lag = 20) and *f*(RH, lag = 20) are modelling nonlinear lag effects of average temperature (TP) and relative humidity (RH), respectively; the rest in equation (1) are used as covariates including time variables, air pollution variables and other meteorological factors. The time smoothing function *f*(*t*) and the categorical variable Year*_t_* are used to control for long-term trend and seasonality. *f*(*t*) is a natural cubic spline (ns) function with respect to the 12 months per year and its degree freedom (df) was six determined based on the minimum Akaike information criterion (AIC)[16]. Spring-Festival*_t_* is a binary variable to control for the potential effects caused by healthcare-seeking delay of TB patients due to their gathering with family members to celebrate this great festival during January or February including the Spring Festival [17]. *τ_t_* is the autoregressive term of the monthly number of TB cases notified in the log scale based on the plot of partial autocorrelation function (Supplementary Fig. S2) to control for the autocorrelations in the residuals [18]. *β*, *γ* and *δ* represent the coefficients of corresponding terms. Offset (log(pop)) is accounted for changes in size of the population using the logarithm of average total population in the annual census data from 1997 to 2018 [19]. We also controlled for the impacts of other meteorological variables by fitting natural cubic splines of average wind (WD) and sunshine duration (s.d.) with *a priori* 3 df, as average temperature was highly corrected with total rainfall (rr = 0.74, *P* < 0.001) and average pressure (rr = 0.92, *P* < 0.001) in our preliminary analysis, we excluded average air pressure and total rainfall from our model to avoid collinearity. In addition, based on preliminary exploratory analyses and findings of previous research [20, 21], PM_10_ and NO*_X_* were modelled using the natural cubic splines with 3 df to control for the effects of air pollution on the number of TB notifications.

We used double natural cubic spline functions to fit the exposure–response relationship and lag effects between meteorological parameters (including average temperature and relative humidity) and the number of TB cases notified. Setting spline knots at equal intervals in the ranges of meteorological measurements was to make enough flexibility in the two ends in the distributions of meteorological parameters; placing spline knots at equal spaces in the scale of lag time was to produce more flexible lag effects at shorter lags [8]. In order to establish a cross-basis function based on the best combination of the two-dimensional (2-D) natural cubic spline functions of both meteorological parameters and lags, we determined the optimum number of knots in each dimension based on the minimum AIC [8, 16]. In the end, based on the minimum AIC, we used the best combination of the cross-basis functions of average temperature, relative humidity and their lags to model the impacts of average temperature and relative humidity on the risk of TB notifications with a maximum lag of 20 months. The final best model showed that the natural cubic spline function of average temperature and its lag time had 3 knots and 5 knots, and the spline of relative humidity and its lag time both had 2 knots.

We used an overall median of average temperature 24.8 °C over the study period as a reference to analyse the exposure–response–lag relationship between average temperature and the number of TB notifications; to explore the exposure–response–lag relationship between relative humidity and the number of TB notifications, we used the relative humidity of 83.7% corresponding to the percentile of a minimum number of TB notifications as the reference. The method for calculating the minimum TB notification percentile was described in the previous study [22]. We plotted three-dimensional (3-D) and contour plots to comprehensively describe the association of average temperature and relative humidity with the risk of TB notifications at different lags with a max lag of 20 months in 1997–2018 in Hong Kong. At the same time, we calculated relative risks (RRs) (95% confidence interval (CI)) of TB notifications at the average temperature with the highest risk *vs.* 24.8 °C at different lagged months. We also calculated the RRs of TB notifications at different lagged months at the relative humidity with the highest risk *vs.* 83.7%. Our data analyses were performed using the dlnm software package in R (version 3.5.0). The positions of weather monitoring stations were mapped using ArcGIS (v.10.5).

## Results

### The distributions of TB notifications, meteorological factors and air pollution

From 1997 to 2018, there were altogether 125, 818 TB notifications in Hong Kong, with an average of 476 ± 108.5 TB notifications per month. During the entire study period, medians of monthly average temperature and relative humidity were 24.8 °C and 80.0%, and monthly medians of PM_10_ and NO*_X_* concentrations were 50.5 and 151.0 μg/m^3^, respectively (Table 1). Monthly average temperature and NO*_X_* were correlated with the total number of TB notifications at lag 0 and other most lags, and monthly relative humidity, average wind, sunshine duration and PM_10_ were correlated with the total number of TB notifications at most lagged months except for lag 0 (Supplementary Table S1).

**Table 1.** Distributions of monthly total TB notifications, meteorological measurements and air pollution in Hong Kong from 1997 to 2018

Variable	Mean ± s.d.	Percentiles				
		P5	P25	P50	P75	P95
Total population (million people/year)	69.8 ± 274.1	65.8	67.3	69.5	72.1	74.1
Total TB cases	477.0 ± 108.5	337.2	392.5	450.5	549.3	681.7
Meteorology measurements						
Mean average temperature (°C)	23.5 ± 4.7	16.1	19.1	24.8	28.0	29.2
Mean relative humidity (%)	78.3 ± 5.6	66.0	75.0	80.0	82.0	85.0
Mean air pressure (hPa)	1012.8 ± 5.6	1004.4	1007.5	1013.2	1017.7	1020.7
Mean wind velocity (m/s)	22.9 ± 3.9	17.1	20.2	22.7	25.8	29.2
Total rainfall (mm/month)	205.2 ± 227.4	3.0	31.8	124.3	319.4	651.3
Sunshine duration (h/month)	151.7 ± 48.3	76.3	116.8	148.5	187.2	226.1
Pollutant concentration						
PM_2.5_ (μg/m^3^)	51.0 ± 17.5	24.2	37.0	50.5	64.0	79.9
PM_10_ (μg/m^3^)	34.0 ± 12.9	14.2	24.0	32.0	44.0	56.0
SO_2_ (μg/m^3^)	15.3 ± 5.8	7.0	10.0	15.0	19.0	25.0
NO_2_ (μg/m^3^)	61.2 ± 12.6	41.0	52.0	61.5	70.0	81.0
CO (mg/m^3^)	0.9 ± 0.2	0.6	0.7	0.8	1.0	1.2
NO*_X_* (μg/m^3^)	148.4 ± 39.0	33.9	127.0	151.0	171.5	230.7

s.d., standard deviation.

*Note*: P5, P25, P50, P75 and P95 are the 5th, 25th, 50th, 75th and 95th percentiles of the variables, respectively.

**Fig. 1.**
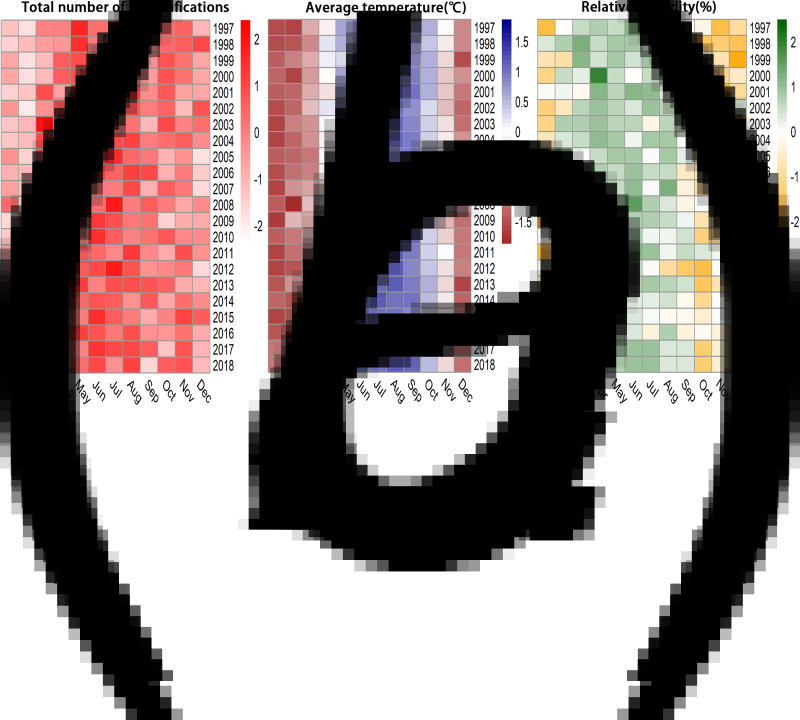
Annual cycle of the total number of TB notifications, average temperature and relative humidity in Hong Kong from 1997 to 2018. Annual cycle of (A) monthly total number of TB notifications; (B) monthly average temperature (°C) averaged over all the weather stations, and (C) monthly relative humidity (%) averaged over all the weather station.

**Fig. 2.**
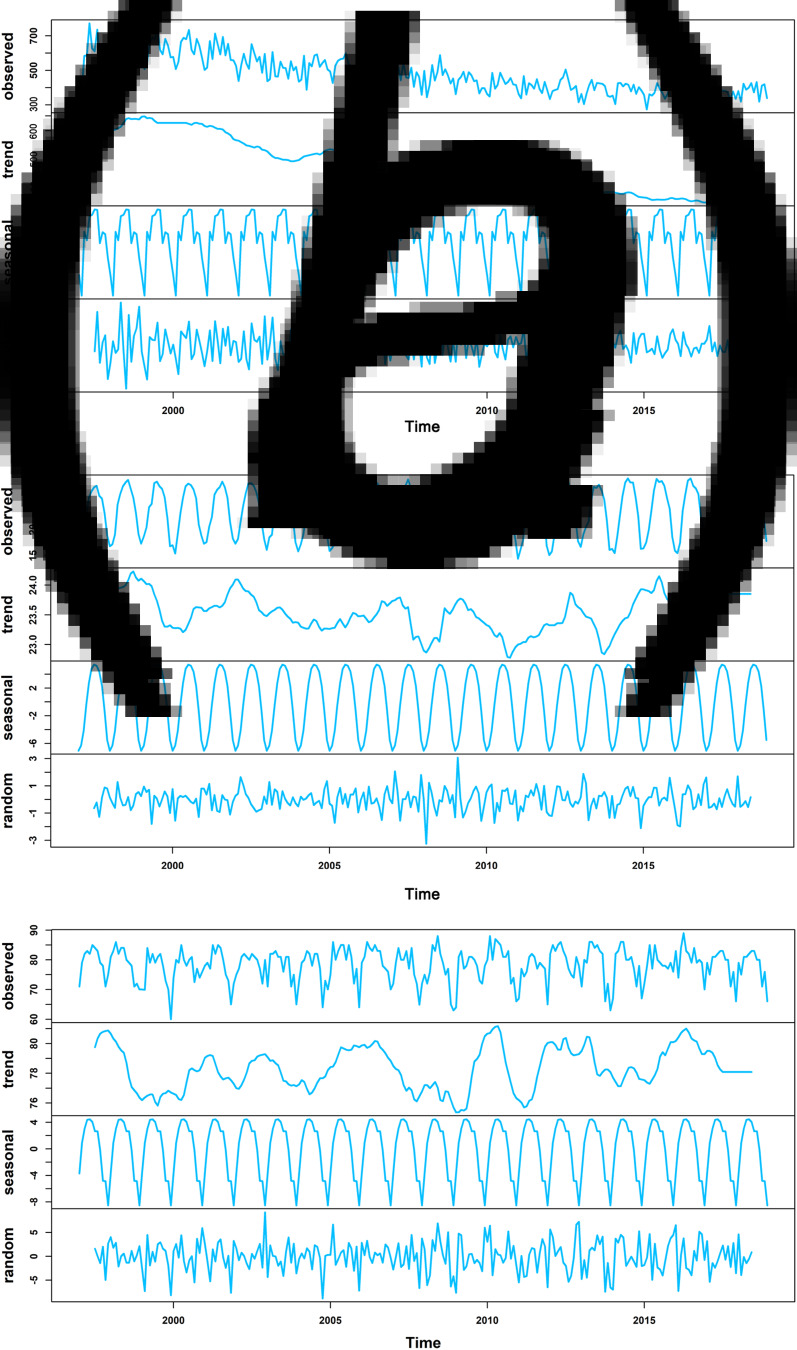
Decomposition plots of the time-series in Hong Kong from 1997 to 2018. The decomposition plot of (A) monthly total number of TB notifications; (B) monthly average temperature, and (C) monthly relative humidity. The top layer shows the original time-series observed. The other layers show the decomposed components, denoting the seasonal component, long term trend component, and remainder component, respectively.

The annual cycles and monthly averages of the total number of TB notifications, average temperature and relative humidity in Hong Kong during the study period are shown in Figure 1 and Supplementary Figure S3. The decomposition results indicated that the number of TB notifications had a decreased trend. It had a seasonal pattern, and the epidemic months were from May to August, followed by March (Fig. 2A and Supplementary Table S2). Average temperature and relative humidity had seasonal distributions, which were prone to have higher average temperature and relative humidity in June–September and March–June, respectively (Fig. 2B, C and Supplementary Tables S3 and S4).

### The exposure–response–lag relationship between TB notifications and meteorological factors and air pollution

**Fig. 3.**
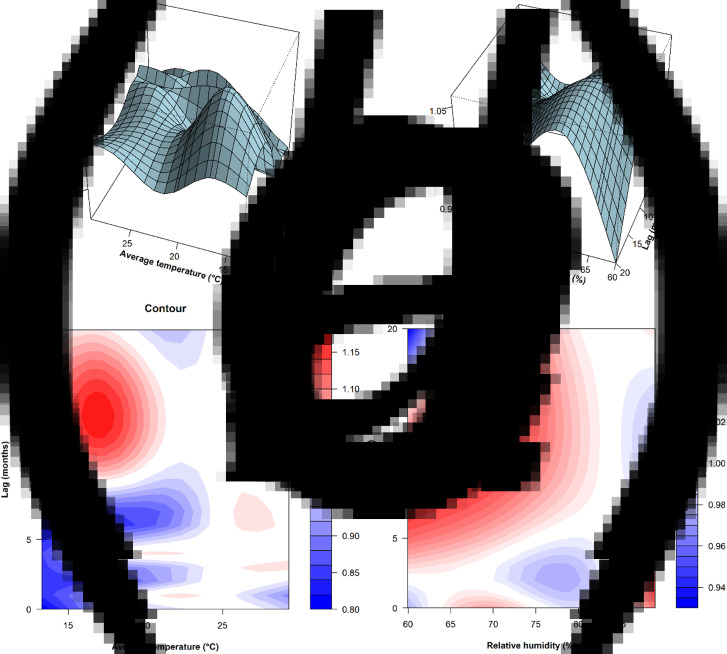
3-D and corresponding contour plots showing the RRs of TB notifications at lagged months along with average temperature and relative humidity in Hong Kong from 1997 to 2018. (A, B) Average temperature (°C) and (C, D) relative humidity (%).

Using average temperature 24.8 °C as a reference, Figure 2A displays a 3-D plot of the estimated effect of monthly average temperature on the number of TB notifications. As this plot indicates, at lower average temperature (*T* < 22.0 °C) and more lagged months (lags ⩾10 months), increases in RRs for TB notifications were noted in Hong Kong. Figure 3B is a contour plot corresponding to the 3-D plot, which identifies the changes of RRs regarding varying average temperatures at different lags with greater clearness. The RRs of TB notifications were greater than 1.15 when average temperature was between 16.3 and 17.3 °C at lags of 13–15 months, and reaches the highest risk of 1.18 (95% CI 1.02–1.35) when it was 16.8 °C at lagged 14 months.

Setting the relative humidity of 83.7% as a reference, Figure 3C depicts how the risk of TB notifications varied with changes in the relative humidity at different lags. As suggested in the 3-D chart, the risk of TB notifications elevated as the relative humidity was <84.0% at lags of over 2 months and ⩾85.0% at lags of 1–6 months. Figure 3D clearly shows that the overall RRs were gradually >1.05 as relative humidity of 60.0–63.6% at lagged 9–11 months expanded to 68.0–71.0% at lagged 12–17 months, reaching the highest risk of 1.06 (95% CI 1.01–1.11) when it was 69.0% at lagged 13 months.

**Fig. 4.**
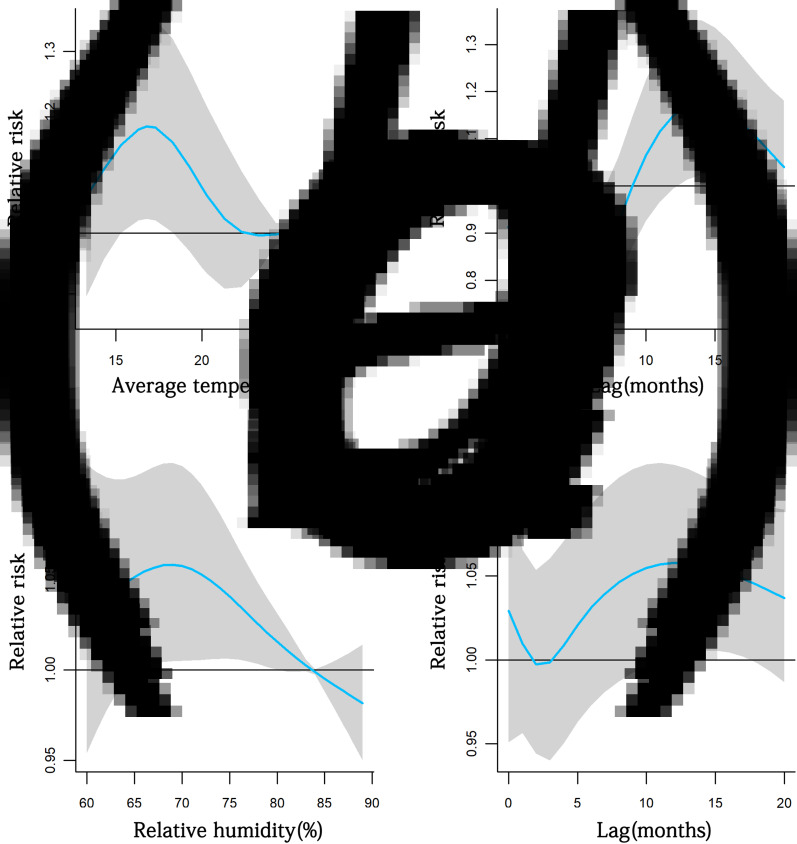
Estimated RRs (95% CI) of TB notifications with (A) average temperatures and (B) relative humidity at selected lagged months (left), and lagged months with respect to selected (C) average temperatures and (D) relative humidity (right) in Hong Kong from 1997 to 2018. Blue solid line: RRs; grey shaded areas: 95% CIs.

In Figure 4A, B and Supplementary Tables S5–S7, the graph on the left depicts the RRs (95% CI) of TB notifications by average temperatures at lagged 14 months, while the graph on the right shows the RRs of TB notifications at different lags at the average temperature of 16.8 °C. In the left graph, increases in the RRs of TB notifications were noted as the average temperature was between 15.3 °C and 18.3 °C at lagged 14 months; in the right graph, increased RRs of TB notifications lasted from lag 12 to lag 17 months as average temperature was 16.8 °C. In Figure 4C, D and Supplementary Tables S6 and S7, the graph on the left depicts the RRs of TB notifications by the relative humidity at lagged 13 months, while the graph on the right shows the RRs of TB notifications at different lags at the relative humidity of 69.0%. On the left graph, increases in the RRs of TB notifications were identified as the relative humidity varied from 66% to 80% at lagged 13 months, while increased RRs of TB notifications lasted from lag 12 month to lag 17 month as relative humidity was 69.0% in the right graph.

## Discussion

Our study demonstrated that average temperature and relative humidity were significant weather-based risk factors of TB notifications in Hong Kong. Furthermore, by feasibly modelling the 2-D exposure–response–lag relationship using the DLNM with a max lag of 20 months, the impacts of these meteorological factors on TB notifications were revealed with their specific magnitudes and lagged time. The temporal lag associations may be helpful for predicting the number of TB notifications in the subsequent months, providing important reference for improving strategies on TB early warning.

*M. tuberculosis* infection may occur when susceptible people inhale the droplet nucleus containing *M. tuberculosis* discharged into the air by active TB patients. Nearly a quarter of the world's population has been infected with *M. tuberculosis*, of whom about 5% can rapidly progress to active TB while there is still chance that a vast majority with latent TB infection may progress to active TB (‘reactivation’) in the future [23]. As TB infection is chronic, endemic and complex by many factors, the observed TB cases notified in Hong Kong in the study period may include both new infection and reactivation. The decline in TB notifications in Hong Kong during the study periods could be accounted for by collaborative efforts of local, national and international institutions and organisations, such as implementing the effective case-finding strategy, increasing the coverage of Directly Observed Treatment Short course, and introducing the TB notification system [24]. The temporal changes of TB notifications in this city indicated obvious seasonality of TB. Its epidemic months were mainly in summer, and the summer peaks of TB were also observed in places of some other countries, such as UK, Iran and Pakistan [25].

Seasonal changes in meteorological conditions played an important role in shaping the temporal variations of TB cases, especially temperature levels [26, 27]. Lower average temperatures were reported to have positive, neutral or negative effects on the numbers of TB cases in places with diverse climates [7, 9, 28, 29]. Our study found that lower average temperatures (<22.0 °C) at more lagged months (>10 months) were associated with increased risks of TB notifications. Particularly, the risks became higher as it varied from 16.3 to 17.3 °C at lags of 13–15 months, and reached the greatest risk when it was 16.8 °C at lagged 14 months. The association of lower average temperature with increased risks of TB cases was also shown in research in Brazil, Bangladesh, Vietnam and mainland China [4, 5, 7, 9, 27]. But to our knowledge, only three studies have considered lag effects of lower temperature on TB cases [7, 9, 27]. The effects of lower average temperature on TB cases occurred at lagged 2 months in Southwest China [7], at a lag of two quarters in Vietnam [27], and at lags of one–to–six quarters in Bangladesh [9]. Various setting of max lags and different time dimensions of data may partially result in the differences in the lags of average temperature observed in above four studies, and other influencing factors need further investigation. One explanatory hypothesis for the effects of lower average temperature is higher chance of transmission with *M. tuberculosis* in the colder months because of long periods of people's indoor activity. The crowded indoor environment and low air flow may provide suitable conditions for the survival and the transmission of *M. tuberculosis* [30]. Moreover, some other factors affecting immunological response that was influenced by the season could also protect the body from the progress of latent infection to the overt symptoms. Among them, a low vitamin D level was evidenced as a potential factor related to impaired host immune resistance accompanying with ‘reactivation’ of *M. tuberculosis* during the incubation [31]. And vitamin D deficiency in winter has been shown to correlate with the peak of TB notifications in summer in Hong Kong [32].

In our study, risks of TB notifications elevated as relative humidity was below 84.0% at lags of 3–20 months and as it was greater than 85.0% at lags of 1–6 months. Higher risks of TB notifications occurred when relative humidity of 60.0–63.6% at lagged 9–11 months expanded to 68.0–71.0% at lagged 12–17 months, reaching the highest risk when it was 69.0% at lagged 13 months. Only two studies observed significant associations of lower humidity with TB cases with its lag effects [7, 27]. A study of Southwest China revealed that minimum relative humidity was inversely correlated with the number of TB cases at lagged 4 months [7]. Data from the National TB programme of Vietnam showed an inverse association between absolute humidity and TB incidence with a lag of two quarters [27]. Besides, lag effects of relative humidity could last for up to six quarters in three known endemic districts of North-East Bangladesh, with immediate higher risks at lower humidity [9]. Although the lags of humidity were inconsistent among these four studies, these findings indicated that a lower level of humidity was closely linked with more TB cases with lag effects of diverse lengths. One possible explanatory reason was that in the dry condition the airway mucus secretion of the body and the clearance of exogenous pathogens were reduced, which may make people more susceptible to *M. tuberculosis* infection [33]. A mice experiment also found that inhalation of dry air damaged airway cilia clearance and tissue repair functions, thereby reducing the body's resistance to pathogens [34]. Some other possible mechanisms may also exist and needs further research. What is more, there may be other reasoned explanations why lower relative humidity triggered the activation of latent TB. In addition, risks of TB notifications were also increased when the relative humidity was >85.0% at lagged 2 months, which was related to patients' feeling sultriness in such high relative humidity, thus accelerating outpatient visits for TB. It is also noteworthy that conclusions between humidity and TB cases in previous studies did not reach an agreement. For example, in the Federal District of Brazil, TB was prevalent with relative humidity between 31.0% and 69.0%, a moderate level, instead of a lower level, of relative humidity for this district [4]. An analysis based on Bayesian theory did not find significant influence of average humidity on the reported TB cases of mainland China [35]. In summary, as TB spread is a result of the interaction of environmental and social factors, the inconsistent conclusions on the associations between average temperature, as well as relative humidity, and TB cases may imply that impacts of meteorological factors on TB cases may be influenced by the confounding effects of some social factors.

Furthermore, the likelihood of infection with *M. tuberculosis* is decided by the number of infectious droplet nuclei per volume of air and the duration to which the susceptible population was exposed [36]. An earlier study proved that *M. tuberculosis* transmission was closely related to the diameter of the droplets containing the pathogens, and their diameters are related to the speed of droplets suspended in the air falling to the ground [37]. In this process, temperature and relative humidity are important factors affecting the formation of the droplet diameters [36]. We found an overlap in the period between lower average temperature and lower relative humidity in winter, which were associated with increased risks of TB notifications. We then speculate that within a certain range of temperature and relative humidity, droplets containing *M. tuberculosis* are more likely to be evaporated into air to form into certain diameters that can be suspended in the air for a longer time, to be easily exhaled into the body by susceptible persons. In addition, lower relative humidity and lower temperature may increase atmosphere surface dust or pollution particulates in the winter, which may attach more pathogenic bacteria including *M. tuberculosis* under certain temperature and humidity conditions [38].

Delays in the diagnosis and treatment of TB patients after the disease onset happen often. A population-based study in Hong Kong revealed that the median of the interval from initial symptoms to initial treatment of TB patients was 49 days (25th–75th percentile: 20–80 days) [11]. Considering that Hong Kong has an advanced medical institution management system and put great emphasis on TB prevention and treatment for a long term, this time interval might be close to the median delay of TB diagnosis in this place. The lag effect of weather factors on the number of TB notifications with the highest risks (Fig. 4) shows that increases in TB notifications lasted from lag 12 months to lag 17 months as average temperatures was 16.8 °C or the relative humidity was 69.0%. A lag of 12–17 months indicates that even given the time error of the delayed diagnosis of TB patients, weather factors may still have lag effects on the pathogenesis of TB, suggesting long-term exposure to specific average temperatures or the relative humidity may be a risk factor in the development of TB in Hong Kong.

The major strength of this study was using a 22-year time-series to study the nonlinear relationship between meteorological factors and the number of TB notifications with DLNM while taking into account a longer incubation period of TB, which fully identified certain magnitudes of meteorological factors with increased risks of TB notifications, as well as their lag effects. Second, we controlled for the effects of air pollution when modelling impacts of meteorological factors on the outcome. Nevertheless, this study has some limitations. First, meteorological indicators and air pollution data used in our research were derived from fixed monitoring stations, so the individual-based association was not allowed. This might introduce measurement error, but the error was likely to be randomly distributed, so the risk estimates may not be attenuated [39]. Second, this study lacked some information on the risk stratification such as age, gender and smoking, and our conclusions may be affected by these confounding factors. Particularly, age dependence may complicate the relationship between meteorological factors and the number of TB cases, which needs further study to explore its possible effect modification. Third, the under-notification of TB was likely to have occurred in Hong Kong as this problem was reported in other parts of China [40]. However, considering the good health care infrastructure, high accessibility of health care, and a comprehensive infectious disease notification system in Hong Kong, the notification rate of TB may be close to the incidence of TB in this area.

## Conclusions

Our study identified non-linear lag effects of average temperature and relative humidity on the number of TB cases notified in Hong Kong. This study may provide preliminary climate-based clues for further research on epidemiological prediction of TB notifications and for the development of early warning systems. However, for more comprehensive understanding of temporal patterns of TB in Hong Kong, the role of non-meteorological variables is a necessary complement to our finding and needs further investigations.

## Data Availability

The data that support the findings of this study are openly available in Zenodo at http://zenodo.org/deposit/4327078, reference number 4327078.

## Supplementary material

For supplementary material accompanying this paper visit https://doi.org/10.1017/S0950268820003040. click here to view supplementary material
