# Supplementary material for: Temperature and humidity associated with increases in tuberculosis notifications: a time-series study in Hong Kong
Source: Epidemiol Infect. 2020 Dec 28;149:e8. doi: 10.1017/S0950268820003040 (PMC8057503; doi:10.1017/S0950268820003040)
Supplement: Supplementary file 1 [file S0950268820003040sup001.docx]

## *Epidemiology and Infection*

Association of temperature and humidity with the number of tuberculosis notifications: a time-series study in Hong Kong

**M. Xu ^1 ,2^, Y. Li ^3^, B. Liu ^4^, R. Chen^5^, L. Sheng ^6^, S. Yan^7^, H. Chen ^8^, J. Hou ^9^, L. Yuan^10^, L. Ke ^4^, M. Fan^1^, P. Hu ^1*^**

## Supplementary materials

 Supplementary Material is available on the Cambridge Core website.

Supplementary Figure S1 The study area and locations of weather stations.

Supplementary Figure S2 The plot of partial auto-correlation function in the distributed lag nonlinear model.

Supplementary Table S1 The spearman rank correlation coefficients between tuberculosis notifications, air pollution and meteorological factors.

Supplementary Figure S3 The averages of monthly total number of tuberculosis notifications, average temperature and relative humidity in Hong Kong from 1997 to 2018.

Supplementary Table S2 The seasonal components of monthly total number of tuberculosis notifications in Hong Kong from 1997 to 2018.

Supplementary Table S3 The seasonal components of monthly average temperature in Hong Kong from 1997 to 2018.

Supplementary Table S4 The seasonal components of monthly relative humidity in Hong Kong from 1997 to 2018.

Supplementary Table S5. Estimated excess risks (95% CI) of tuberculosis notifications with average temperatures at lagged 14 months in Hong Kong from 1997 to 2018.

Supplementary Table S6. Estimated excess risks (95% CI) of tuberculosis notifications with Relative humidity at lagged 13 months in Hong Kong from 1997 to 2018.

Supplementary Table S7. Estimated excess risks (95% CI) of tuberculosis notifications with average temperatures (16.8 ℃) and relative humidity (69%) at lagged months in Hong Kong from 1997 to 2018.


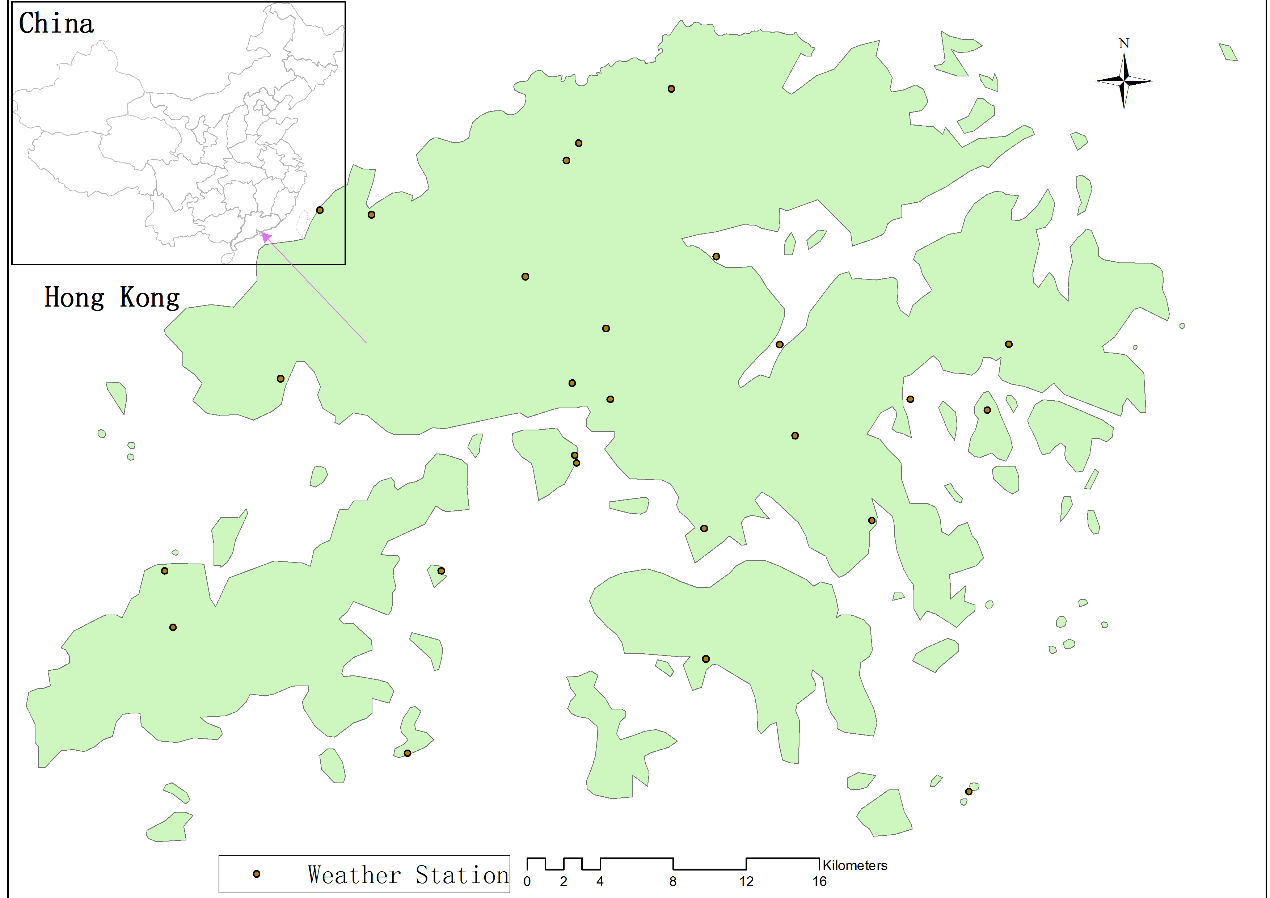
Supplementary Figure S1. The study area and locations of weather stations.


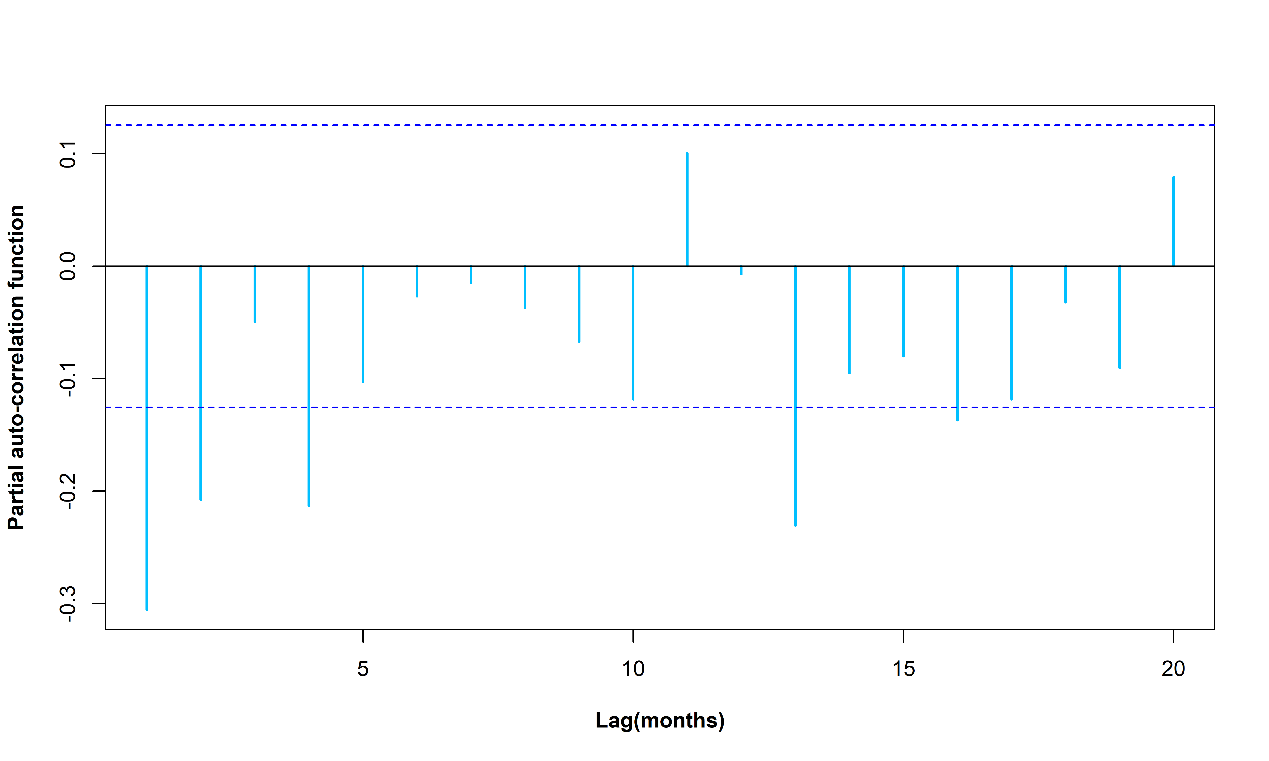


 Supplementary Figure S2. The plot of partial auto-correlation function in the distributed lag nonlinear model.

Supplementary Table S1. The spearman rank correlation coefficients between tuberculosis notifications, air pollution and meteorological factors

| Variable | TP | RH | WD | SD | RF | PR | PM_10_ | NO_X_ | Lag 0 |
| --- | --- | --- | --- | --- | --- | --- | --- | --- | --- |
| TP | 1 | 0.31^**^ | -0.52^**^ | 0.42^**^ | 0.74^**^ | -0.92^**^ | -0.66^**^ | -0.41^**^ | 0.25^*^ |
| RH |  | 1 | -0.41^**^ | -0.56^**^ | 0.61^**^ | -0.52^**^ | -0.58^**^ | -0.16 | 0.11 |
| WD |  |  | 1 | -0.10 | -0.43^**^ | 0.54^**^ | 0.37^**^ | 0.02 | -0.11 |
| SD |  |  |  | 1 | -0.05 | -0.19 | 0.03 | -0.10 | 0.04 |
| RF |  |  |  |  | 1 | -0.83^**^ | -0.69^**^ | -0.30^**^ | 0.23^*^ |
| PR |  |  |  |  |  | 1 | 0.68^**^ | 0.30^**^ | -0.30^**^ |
| PM_10_ |  |  |  |  |  |  | 1 | 0.63^**^ | 0.16 |
| NO_X_ |  |  |  |  |  |  |  | 1 | 0.48^**^ |
| Lag 0 |  |  |  |  |  |  |  |  | 1 |
| Lag 1 | 0.23^**^ | -0.04 | -0.04 | 0.17^*^ | 0.18^**^ | -0.24^**^ | 0.22^**^ | 0.48^**^ | 0.78^**^ |
| Lag 2 | 0.16^*^ | -0.13^*^ | -0.05 | 0.21^**^ | 0.10 | -0.16^*^ | 0.27^**^ | 0.54^**^ | 0.78^**^ |
| Lag 3 | 0.03 | -0.21^**^ | 0.08 | 0.16^*^ | -0.04 | -0.02 | 0.38^**^ | 0.60^**^ | 0.75^**^ |
| Lag 4 | -0.10 | -0.27^**^ | 0.15^*^ | 0.15^*^ | -0.19^**^ | 0.12 | 0.50^**^ | 0.65^**^ | 0.67^**^ |
| Lag 5 | -0.22^**^ | -0.23^**^ | 0.15^*^ | 0.03 | -0.22^**^ | 0.21^**^ | 0.58^**^ | 0.71^**^ | 0.68^**^ |
| Lag 6 | -0.29^**^ | -0.2^**^ | 0.16^*^ | -0.06 | -0.26^**^ | 0.26^**^ | 0.57^**^ | 0.72^**^ | 0.66^**^ |

Abbreviations：TP: Temperature; RH：Relative Humidity; WD: Wind; SD: Sunshine Duration; PR: Pressure; RF: Rainfall. Lag 0-lag 20: counts of tuberculosis cases notified from lagged 0 month to lagged 20 months.

^*^*P* <0.05, ^**^*P* <0.05.

Supplementary Table S1 Continued. The spearman rank correlation coefficients between tuberculosis notifications, air pollution and meteorological factors

| Variable | TP | RH | WD | SD | RF | PR | PM_10_ | NO_X_ | Lag 0 |
| --- | --- | --- | --- | --- | --- | --- | --- | --- | --- |
| Lag 7 | -0.28^**^ | -0.10 | 0.13^*^ | -0.12 | -0.24^**^ | 0.21^**^ | 0.49^**^ | 0.71^**^ | 0.65^**^ |
| Lag 8 | -0.20^**^ | 0.02 | -0.03 | -0.15^*^ | -0.11 | 0.10 | 0.41^**^ | 0.75^**^ | 0.70^**^ |
| Lag 9 | -0.08 | 0.13^*^ | -0.10 | -0.15^*^ | 0.02 | -0.04 | 0.30^**^ | 0.70^**^ | 0.71^**^ |
| Lag 10 | 0.07 | 0.17^*^ | -0.19^**^ | -0.09 | 0.15^*^ | -0.19^**^ | 0.22^**^ | 0.62^**^ | 0.75^**^ |
| Lag 11 | 0.20^**^ | 0.18^**^ | -0.19^**^ | -0.01 | 0.19^**^ | -0.28^**^ | 0.16^*^ | 0.54^**^ | 0.81^**^ |
| Lag 12 | 0.26^**^ | 0.08 | -0.10 | 0.12 | 0.21^**^ | -0.29^**^ | 0.15^*^ | 0.50^**^ | 0.83^**^ |
| Lag 13 | 0.24^**^ | -0.03 | -0.11 | 0.19^**^ | 0.18^**^ | -0.26^**^ | 0.20^**^ | 0.53^**^ | 0.76^**^ |
| Lag 14 | 0.14^*^ | -0.12 | -0.01 | 0.23^**^ | 0.08 | -0.15^*^ | 0.28^**^ | 0.57^**^ | 0.77^**^ |
| Lag 15 | 0.02 | -0.22^**^ | 0.11 | 0.18^**^ | -0.06 | -0.02 | 0.40^**^ | 0.62^**^ | 0.69^**^ |
| Lag 16 | -0.11 | -0.24^**^ | 0.10 | 0.15^*^ | -0.17^*^ | 0.12 | 0.51^**^ | 0.70^**^ | 0.66^**^ |
| Lag 17 | -0.25^**^ | -0.24^**^ | 0.12 | 0.03 | -0.22^**^ | 0.21^**^ | 0.60^**^ | 0.75^**^ | 0.66^**^ |
| Lag 18 | -0.30^**^ | -0.19^**^ | 0.16^*^ | -0.07 | -0.25^**^ | 0.26^**^ | 0.59^**^ | 0.74^**^ | 0.6^**^ |
| Lag 19 | -0.30^**^ | -0.08 | 0.10 | -0.12 | -0.21^**^ | 0.22^**^ | 0.50^**^ | 0.74^**^ | 0.63^**^ |
| Lag 20 | -0.21^**^ | 0.03 | -0.02 | -0.14^*^ | -0.13 | 0.09 | 0.41^**^ | 0.74^**^ | 0.70^**^ |

Abbreviations：TP: Temperature; RH：Relative Humidity; WD: Wind; SD: Sunshine Duration; PR: Pressure; RF: Rainfall. Lag 0-lag 20: counts of tuberculosis cases notified from lagged 0 month to lagged 20 months.

^*^*P* <0.05, ^**^*P* <0.05.


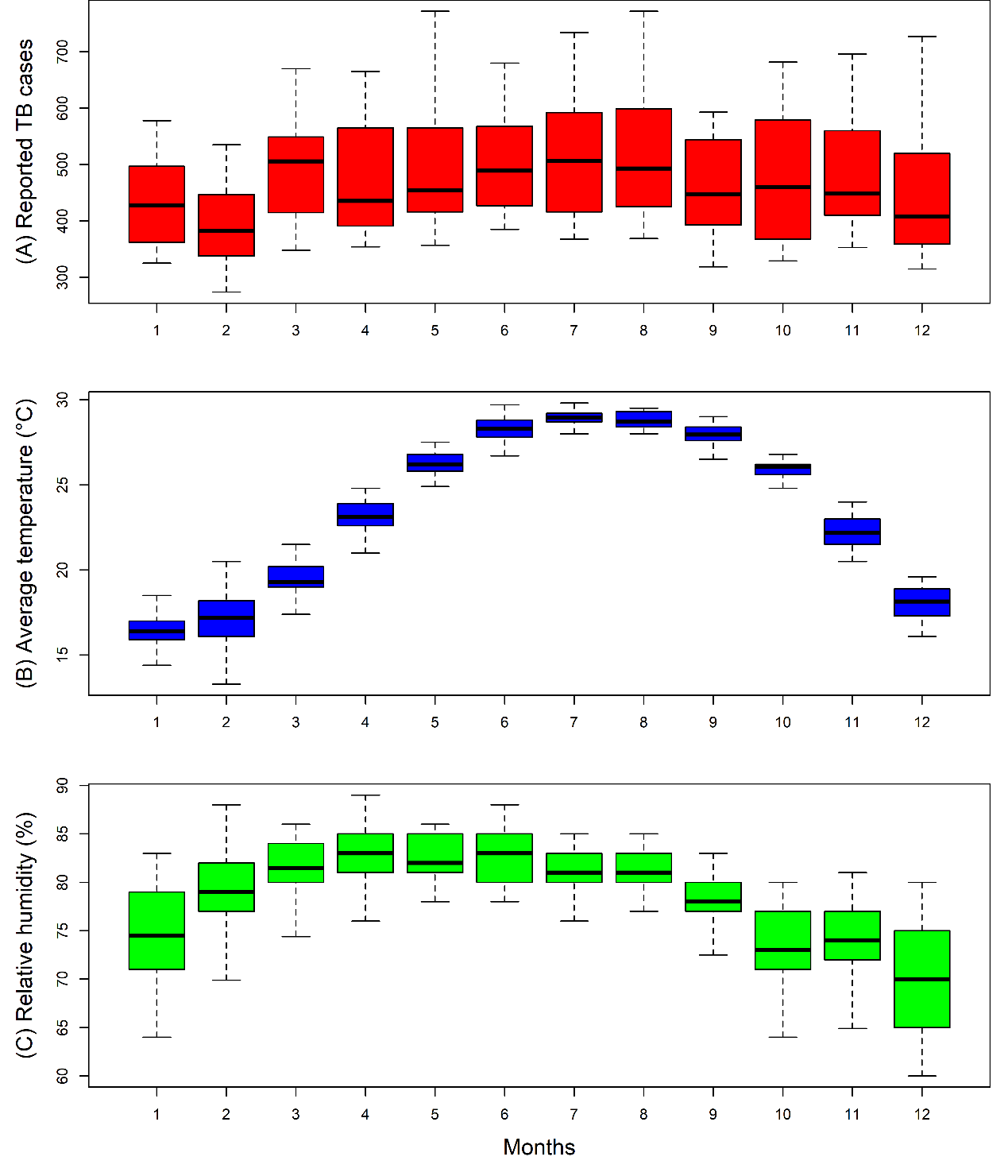


Supplementary Figure S3. The averages of monthly total number of tuberculosis notifications, average temperature and relative humidity in Hong Kong from 1997 to 2018. The averages of (A) monthly total number of tuberculosis notifications; (B) monthly average temperature (°C) averaged over all the weather stations; and (C) monthly relative humidity (%) averaged over all the weather station.

Supplementary Table S2. The seasonal components of monthly total number of tuberculosis notifications in Hong Kong from 1997 to 2018.

| Year | Jan | Feb | Mar | Apr | May | Jun | Jul | Aug | Sep | Oct | Nov | Dec |
| --- | --- | --- | --- | --- | --- | --- | --- | --- | --- | --- | --- | --- |
| 1997 | -50.5 | -78.7619 | 9.047619 | -2.75794 | 30.61706 | 32.375 | 39.78571 | 39.20238 | -6.63492 | 8.456349 | 5.634921 | -26.4643 |
| 1998 | -50.5 | -78.7619 | 9.047619 | -2.75794 | 30.61706 | 32.375 | 39.78571 | 39.20238 | -6.63492 | 8.456349 | 5.634921 | -26.4643 |
| 1999 | -50.5 | -78.7619 | 9.047619 | -2.75794 | 30.61706 | 32.375 | 39.78571 | 39.20238 | -6.63492 | 8.456349 | 5.634921 | -26.4643 |
| 2000 | -50.5 | -78.7619 | 9.047619 | -2.75794 | 30.61706 | 32.375 | 39.78571 | 39.20238 | -6.63492 | 8.456349 | 5.634921 | -26.4643 |
| 2001 | -50.5 | -78.7619 | 9.047619 | -2.75794 | 30.61706 | 32.375 | 39.78571 | 39.20238 | -6.63492 | 8.456349 | 5.634921 | -26.4643 |
| 2002 | -50.5 | -78.7619 | 9.047619 | -2.75794 | 30.61706 | 32.375 | 39.78571 | 39.20238 | -6.63492 | 8.456349 | 5.634921 | -26.4643 |
| 2003 | -50.5 | -78.7619 | 9.047619 | -2.75794 | 30.61706 | 32.375 | 39.78571 | 39.20238 | -6.63492 | 8.456349 | 5.634921 | -26.4643 |
| 2004 | -50.5 | -78.7619 | 9.047619 | -2.75794 | 30.61706 | 32.375 | 39.78571 | 39.20238 | -6.63492 | 8.456349 | 5.634921 | -26.4643 |
| 2005 | -50.5 | -78.7619 | 9.047619 | -2.75794 | 30.61706 | 32.375 | 39.78571 | 39.20238 | -6.63492 | 8.456349 | 5.634921 | -26.4643 |
| 2006 | -50.5 | -78.7619 | 9.047619 | -2.75794 | 30.61706 | 32.375 | 39.78571 | 39.20238 | -6.63492 | 8.456349 | 5.634921 | -26.4643 |
| 2007 | -50.5 | -78.7619 | 9.047619 | -2.75794 | 30.61706 | 32.375 | 39.78571 | 39.20238 | -6.63492 | 8.456349 | 5.634921 | -26.4643 |
| 2008 | -50.5 | -78.7619 | 9.047619 | -2.75794 | 30.61706 | 32.375 | 39.78571 | 39.20238 | -6.63492 | 8.456349 | 5.634921 | -26.4643 |
| 2009 | -50.5 | -78.7619 | 9.047619 | -2.75794 | 30.61706 | 32.375 | 39.78571 | 39.20238 | -6.63492 | 8.456349 | 5.634921 | -26.4643 |
| 2010 | -50.5 | -78.7619 | 9.047619 | -2.75794 | 30.61706 | 32.375 | 39.78571 | 39.20238 | -6.63492 | 8.456349 | 5.634921 | -26.4643 |
| 2011 | -50.5 | -78.7619 | 9.047619 | -2.75794 | 30.61706 | 32.375 | 39.78571 | 39.20238 | -6.63492 | 8.456349 | 5.634921 | -26.4643 |
| 2012 | -50.5 | -78.7619 | 9.047619 | -2.75794 | 30.61706 | 32.375 | 39.78571 | 39.20238 | -6.63492 | 8.456349 | 5.634921 | -26.4643 |
| 2013 | -50.5 | -78.7619 | 9.047619 | -2.75794 | 30.61706 | 32.375 | 39.78571 | 39.20238 | -6.63492 | 8.456349 | 5.634921 | -26.4643 |
| 2014 | -50.5 | -78.7619 | 9.047619 | -2.75794 | 30.61706 | 32.375 | 39.78571 | 39.20238 | -6.63492 | 8.456349 | 5.634921 | -26.4643 |
| 2015 | -50.5 | -78.7619 | 9.047619 | -2.75794 | 30.61706 | 32.375 | 39.78571 | 39.20238 | -6.63492 | 8.456349 | 5.634921 | -26.4643 |

Supplementary Table S2 Continued. The seasonal components of monthly total number of tuberculosis notifications in Hong Kong from 1997 to 2018.

| Year | Jan | Feb | Mar | Apr | May | Jun | Jul | Aug | Sep | Oct | Nov | Dec |
| --- | --- | --- | --- | --- | --- | --- | --- | --- | --- | --- | --- | --- |
| 2016 | -50.5 | -78.7619 | 9.047619 | -2.75794 | 30.61706 | 32.375 | 39.78571 | 39.20238 | -6.63492 | 8.456349 | 5.634921 | -26.4643 |
| 2017 | -50.5 | -78.7619 | 9.047619 | -2.75794 | 30.61706 | 32.375 | 39.78571 | 39.20238 | -6.63492 | 8.456349 | 5.634921 | -26.4643 |
| 2018 | -50.5 | -78.7619 | 9.047619 | -2.75794 | 30.61706 | 32.375 | 39.78571 | 39.20238 | -6.63492 | 8.456349 | 5.634921 | -26.4643 |

Supplementary Table S3. The seasonal components of monthly average temperature in Hong Kong from 1997 to 2018.

| Year | Jan | Feb | Mar | Apr | May | Jun | Jul | Aug | Sep | Oct | Nov | Dec |
| --- | --- | --- | --- | --- | --- | --- | --- | --- | --- | --- | --- | --- |
| 1997 | -7.05456 | -6.31468 | -4.16429 | -0.43194 | 2.711905 | 4.777778 | 5.400992 | 5.228968 | 4.390675 | 2.310318 | -1.33333 | -5.52183 |
| 1998 | -7.05456 | -6.31468 | -4.16429 | -0.43194 | 2.711905 | 4.777778 | 5.400992 | 5.228968 | 4.390675 | 2.310318 | -1.33333 | -5.52183 |
| 1999 | -7.05456 | -6.31468 | -4.16429 | -0.43194 | 2.711905 | 4.777778 | 5.400992 | 5.228968 | 4.390675 | 2.310318 | -1.33333 | -5.52183 |
| 2000 | -7.05456 | -6.31468 | -4.16429 | -0.43194 | 2.711905 | 4.777778 | 5.400992 | 5.228968 | 4.390675 | 2.310318 | -1.33333 | -5.52183 |
| 2001 | -7.05456 | -6.31468 | -4.16429 | -0.43194 | 2.711905 | 4.777778 | 5.400992 | 5.228968 | 4.390675 | 2.310318 | -1.33333 | -5.52183 |
| 2002 | -7.05456 | -6.31468 | -4.16429 | -0.43194 | 2.711905 | 4.777778 | 5.400992 | 5.228968 | 4.390675 | 2.310318 | -1.33333 | -5.52183 |
| 2003 | -7.05456 | -6.31468 | -4.16429 | -0.43194 | 2.711905 | 4.777778 | 5.400992 | 5.228968 | 4.390675 | 2.310318 | -1.33333 | -5.52183 |
| 2004 | -7.05456 | -6.31468 | -4.16429 | -0.43194 | 2.711905 | 4.777778 | 5.400992 | 5.228968 | 4.390675 | 2.310318 | -1.33333 | -5.52183 |
| 2005 | -7.05456 | -6.31468 | -4.16429 | -0.43194 | 2.711905 | 4.777778 | 5.400992 | 5.228968 | 4.390675 | 2.310318 | -1.33333 | -5.52183 |
| 2006 | -7.05456 | -6.31468 | -4.16429 | -0.43194 | 2.711905 | 4.777778 | 5.400992 | 5.228968 | 4.390675 | 2.310318 | -1.33333 | -5.52183 |
| 2007 | -7.05456 | -6.31468 | -4.16429 | -0.43194 | 2.711905 | 4.777778 | 5.400992 | 5.228968 | 4.390675 | 2.310318 | -1.33333 | -5.52183 |
| 2008 | -7.05456 | -6.31468 | -4.16429 | -0.43194 | 2.711905 | 4.777778 | 5.400992 | 5.228968 | 4.390675 | 2.310318 | -1.33333 | -5.52183 |
| 2009 | -7.05456 | -6.31468 | -4.16429 | -0.43194 | 2.711905 | 4.777778 | 5.400992 | 5.228968 | 4.390675 | 2.310318 | -1.33333 | -5.52183 |
| 2010 | -7.05456 | -6.31468 | -4.16429 | -0.43194 | 2.711905 | 4.777778 | 5.400992 | 5.228968 | 4.390675 | 2.310318 | -1.33333 | -5.52183 |
| 2011 | -7.05456 | -6.31468 | -4.16429 | -0.43194 | 2.711905 | 4.777778 | 5.400992 | 5.228968 | 4.390675 | 2.310318 | -1.33333 | -5.52183 |
| 2012 | -7.05456 | -6.31468 | -4.16429 | -0.43194 | 2.711905 | 4.777778 | 5.400992 | 5.228968 | 4.390675 | 2.310318 | -1.33333 | -5.52183 |
| 2013 | -7.05456 | -6.31468 | -4.16429 | -0.43194 | 2.711905 | 4.777778 | 5.400992 | 5.228968 | 4.390675 | 2.310318 | -1.33333 | -5.52183 |
| 2014 | -7.05456 | -6.31468 | -4.16429 | -0.43194 | 2.711905 | 4.777778 | 5.400992 | 5.228968 | 4.390675 | 2.310318 | -1.33333 | -5.52183 |
| 2015 | -7.05456 | -6.31468 | -4.16429 | -0.43194 | 2.711905 | 4.777778 | 5.400992 | 5.228968 | 4.390675 | 2.310318 | -1.33333 | -5.52183 |

Supplementary Table S3 continued. The seasonal components of monthly average temperature in Hong Kong from 1997 to 2018.

| Year | Jan | Feb | Mar | Apr | May | Jun | Jul | Aug | Sep | Oct | Nov | Dec |
| --- | --- | --- | --- | --- | --- | --- | --- | --- | --- | --- | --- | --- |
| 2016 | -7.05456 | -6.31468 | -4.16429 | -0.43194 | 2.711905 | 4.777778 | 5.400992 | 5.228968 | 4.390675 | 2.310318 | -1.33333 | -5.52183 |
| 2017 | -7.05456 | -6.31468 | -4.16429 | -0.43194 | 2.711905 | 4.777778 | 5.400992 | 5.228968 | 4.390675 | 2.310318 | -1.33333 | -5.52183 |
| 2018 | -7.05456 | -6.31468 | -4.16429 | -0.43194 | 2.711905 | 4.777778 | 5.400992 | 5.228968 | 4.390675 | 2.310318 | -1.33333 | -5.52183 |

Supplementary Table S4. The seasonal components of monthly relative humidity in Hong Kong from 1997 to 2018.

| Year | Jan | Feb | Mar | Apr | May | Jun | Jul | Aug | Sep | Oct | Nov | Dec |
| --- | --- | --- | --- | --- | --- | --- | --- | --- | --- | --- | --- | --- |
| 1997 | -3.70169 | 0.872917 | 3.043552 | 4.369742 | 4.468948 | 4.048314 | 2.657441 | 2.649504 | -0.22153 | -4.81081 | -4.85923 | -8.51716 |
| 1998 | -3.70169 | 0.872917 | 3.043552 | 4.369742 | 4.468948 | 4.048314 | 2.657441 | 2.649504 | -0.22153 | -4.81081 | -4.85923 | -8.51716 |
| 1999 | -3.70169 | 0.872917 | 3.043552 | 4.369742 | 4.468948 | 4.048314 | 2.657441 | 2.649504 | -0.22153 | -4.81081 | -4.85923 | -8.51716 |
| 2000 | -3.70169 | 0.872917 | 3.043552 | 4.369742 | 4.468948 | 4.048314 | 2.657441 | 2.649504 | -0.22153 | -4.81081 | -4.85923 | -8.51716 |
| 2001 | -3.70169 | 0.872917 | 3.043552 | 4.369742 | 4.468948 | 4.048314 | 2.657441 | 2.649504 | -0.22153 | -4.81081 | -4.85923 | -8.51716 |
| 2002 | -3.70169 | 0.872917 | 3.043552 | 4.369742 | 4.468948 | 4.048314 | 2.657441 | 2.649504 | -0.22153 | -4.81081 | -4.85923 | -8.51716 |
| 2003 | -3.70169 | 0.872917 | 3.043552 | 4.369742 | 4.468948 | 4.048314 | 2.657441 | 2.649504 | -0.22153 | -4.81081 | -4.85923 | -8.51716 |
| 2004 | -3.70169 | 0.872917 | 3.043552 | 4.369742 | 4.468948 | 4.048314 | 2.657441 | 2.649504 | -0.22153 | -4.81081 | -4.85923 | -8.51716 |
| 2005 | -3.70169 | 0.872917 | 3.043552 | 4.369742 | 4.468948 | 4.048314 | 2.657441 | 2.649504 | -0.22153 | -4.81081 | -4.85923 | -8.51716 |
| 2006 | -3.70169 | 0.872917 | 3.043552 | 4.369742 | 4.468948 | 4.048314 | 2.657441 | 2.649504 | -0.22153 | -4.81081 | -4.85923 | -8.51716 |
| 2007 | -3.70169 | 0.872917 | 3.043552 | 4.369742 | 4.468948 | 4.048314 | 2.657441 | 2.649504 | -0.22153 | -4.81081 | -4.85923 | -8.51716 |
| 2008 | -3.70169 | 0.872917 | 3.043552 | 4.369742 | 4.468948 | 4.048314 | 2.657441 | 2.649504 | -0.22153 | -4.81081 | -4.85923 | -8.51716 |
| 2009 | -3.70169 | 0.872917 | 3.043552 | 4.369742 | 4.468948 | 4.048314 | 2.657441 | 2.649504 | -0.22153 | -4.81081 | -4.85923 | -8.51716 |
| 2010 | -3.70169 | 0.872917 | 3.043552 | 4.369742 | 4.468948 | 4.048314 | 2.657441 | 2.649504 | -0.22153 | -4.81081 | -4.85923 | -8.51716 |
| 2011 | -3.70169 | 0.872917 | 3.043552 | 4.369742 | 4.468948 | 4.048314 | 2.657441 | 2.649504 | -0.22153 | -4.81081 | -4.85923 | -8.51716 |
| 2012 | -3.70169 | 0.872917 | 3.043552 | 4.369742 | 4.468948 | 4.048314 | 2.657441 | 2.649504 | -0.22153 | -4.81081 | -4.85923 | -8.51716 |
| 2013 | -3.70169 | 0.872917 | 3.043552 | 4.369742 | 4.468948 | 4.048314 | 2.657441 | 2.649504 | -0.22153 | -4.81081 | -4.85923 | -8.51716 |
| 2014 | -3.70169 | 0.872917 | 3.043552 | 4.369742 | 4.468948 | 4.048314 | 2.657441 | 2.649504 | -0.22153 | -4.81081 | -4.85923 | -8.51716 |
| 2015 | -3.70169 | 0.872917 | 3.043552 | 4.369742 | 4.468948 | 4.048314 | 2.657441 | 2.649504 | -0.22153 | -4.81081 | -4.85923 | -8.51716 |

Supplementary Table S4 continued. The seasonal components of monthly relative humidity in Hong Kong from 1997 to 2018.

| Year | Jan | Feb | Mar | Apr | May | Jun | Jul | Aug | Sep | Oct | Nov | Dec |
| --- | --- | --- | --- | --- | --- | --- | --- | --- | --- | --- | --- | --- |
| 2016 | -3.70169 | 0.872917 | 3.043552 | 4.369742 | 4.468948 | 4.048314 | 2.657441 | 2.649504 | -0.22153 | -4.81081 | -4.85923 | -8.51716 |
| 2017 | -3.70169 | 0.872917 | 3.043552 | 4.369742 | 4.468948 | 4.048314 | 2.657441 | 2.649504 | -0.22153 | -4.81081 | -4.85923 | -8.51716 |
| 2018 | -3.70169 | 0.872917 | 3.043552 | 4.369742 | 4.468948 | 4.048314 | 2.657441 | 2.649504 | -0.22153 | -4.81081 | -4.85923 | -8.51716 |

Supplementary Table S5. Estimated excess risks (95% CI) of tuberculosis notifications with average temperatures (16.8 ℃) and relative humidity (69%) at lagged months in Hong Kong from 1997 to 2018.

| Lag | Average temperature (℃) | | | Relative humidity (%) | | | | | | |
| --- | --- | --- | --- | --- | --- | --- | --- | --- | --- | --- |
|  | ER | Lower limit | Up limit | | ER | Lower limit | | Up limit | |  |
| Lag 0 | -8.79 | -21.46 | 5.92 | | 3.06 | | -4.6 | | 11.32 | |
| Lag 1 | -8.35 | -19.91 | 4.89 | | 0.93 | | 5 | | 6.5 | |
| Lag 2 | -14.63 | -26.37 | -1.01 | | -0.39 | | -5.77 | | 5.31 | |
| Lag 3 | -10.03 | -22.6 | 4.6 | | -0.35 | | -6.26 | | 5.94 | |
| Lag 4 | -1.61 | -14.84 | 13.68 | | 0.64 | | -5.29 | | 6.94 | |
| Lag 5 | -9.5 | -21.4 | 4.21 | | 1.82 | | -4.03 | | 8.02 | |
| Lag 6 | -16.15 | -27.9 | -2.47 | | 2.84 | | -3.01 | | 9.04 | |
| Lag 7 | -14.42 | -26.4 | -0.48 | | 3.69 | | -2.19 | | 9.93 | |
| Lag 8 | -7.78 | -19.94 | 6.22 | | 4.39 | | -1.52 | | 10.64 | |
| Lag 9 | -0.03 | -12.87 | 14.7 | | 4.93 | | -0.96 | | 11.16 | |
| Lag 10 | 6.48 | -7.42 | 22.47 | | 5.33 | | -0.49 | | 11.48 | |
| Lag 11 | 11.5 | -3.4 | 28.69 | | 5.6 | | -0.09 | | 11.62 | |
| Lag 12 | 14.98 | -0.53 | 32.92 | | 5.75 | | 0.23 | | 11.59 | |
| Lag 13 | 16.98 | 1.34 | 35.02 | | 5.8 | | 0.48 | | 11.42 | |
| Lag 14 | 17.59 | 2.33 | 35.14 | | 5.76 | | 0.64 | | 11.15 | |
| Lag 15 | 17.02 | 2.49 | 33.6 | | 5.65 | | 0.72 | | 10.81 | |

ER: excess risk.

Supplementary Table S5 Continued. Estimated excess risks (95% CI) of tuberculosis notifications with average temperatures (16.8 ℃) and relative humidity (69%) at lagged months in Hong Kong from 1997 to 2018.

| Lag | Average temperature (℃) | | | Relative humidity (%) | | | | | | |
| --- | --- | --- | --- | --- | --- | --- | --- | --- | --- | --- |
|  | ER | Lower limit | Up limit | | ER | Lower limit | | Up limit | |  |
| Lag 16 | 15.45 | 1.85 | 30.87 | | 5.46 | | 0.68 | | 10.47 | |
| Lag 17 | 13.14 | 0.41 | 27.48 | | 5.23 | | 0.52 | | 10.15 | |
| Lag 18 | 10.31 | -1.84 | 23.96 | | 4.95 | | 0.23 | | 9.89 | |
| Lag 19 | 7.19 | -4.84 | 20.74 | | 4.65 | | -0.19 | | 9.73 | |
| Lag 20 | 3.98 | -8.43 | 18.06 | | 4.34 | | -0.73 | | 9.67 | |

ER: excess risk.

Supplementary Table S6. Estimated excess risks (95% CI) of tuberculosis notifications with average temperatures at lagged 14 months in Hong Kong from 1997 to 2018.

| Average temperature (℃) | Excess risk | Lower limit | Up limit |
| --- | --- | --- | --- |
| 13.3 | 13.29 | 0.36 | 27.88 |
| 14.3 | 15.44 | 1.85 | 30.83 |
| 15.3 | 17.36 | 2.89 | 33.87 |
| 16.3 | 18.78 | 3.5 | 36.31 |
| 16.8 | 19.21 | 3.67 | 37.07 |
| 17.3 | 19.4 | 3.75 | 37.41 |
| 18.3 | 18.95 | 3.68 | 36.46 |
| 19.3 | 17.18 | 3.31 | 32.91 |
| 20.0 | 15.22 | 2.9 | 29.02 |
| 20.3 | 14.26 | 2.7 | 27.12 |
| 21.3 | 10.71 | 1.94 | 20.23 |
| 22.3 | 7.02 | 1.14 | 13.25 |
| 23.3 | 3.65 | 0.43 | 6.98 |
| 24.3 | 0.98 | 0 | 1.96 |
| 25.3 | -0.74 | -1.66 | 0.18 |
| 26.3 | -1.64 | -4.35 | 1.16 |
| 27.3 | -1.9 | -6.55 | 2.97 |
| 28.3 | -1.72 | -8.48 | 5.54 |
| 29.3 | -1.28 | -10.31 | 8.66 |

Supplementary Table S7. Estimated excess risks (95% CI) of tuberculosis notifications with Relative humidity at lagged 13 months in Hong Kong from 1997 to 2018.

| Relative humidity (%) | Excess risk | Lower limit | Up limit |
| --- | --- | --- | --- |
| 60 | 2.84 | -4.66 | 10.94 |
| 61 | 3.29 | -3.49 | 10.55 |
| 62 | 3.73 | -2.42 | 10.28 |
| 63 | 4.15 | -1.52 | 10.15 |
| 64 | 4.54 | -0.8 | 10.17 |
| 65 | 4.88 | -0.3 | 10.32 |
| 66 | 5.16 | 0.02 | 10.56 |
| 67 | 5.37 | 0.2 | 10.81 |
| 68 | 5.51 | 0.28 | 11 |
| 69 | 5.54 | 0.32 | 11.04 |
| 70 | 5.47 | 0.34 | 10.87 |
| 71 | 5.3 | 0.37 | 10.47 |
| 72 | 5.03 | 0.41 | 9.86 |
| 73 | 4.69 | 0.46 | 9.09 |
| 74 | 4.28 | 0.5 | 8.21 |
| 75 | 3.83 | 0.51 | 7.26 |
| 76 | 3.35 | 0.49 | 6.29 |
| 77 | 2.85 | 0.42 | 5.35 |
| 78 | 2.36 | 0.3 | 4.46 |

Supplementary Table S7 continued. Estimated excess risks (95% CI) of tuberculosis notifications with Relative humidity at lagged 13 months in Hong Kong from 1997 to 2018.

| Relative humidity (%) | Excess risk | Lower limit | Up limit |
| --- | --- | --- | --- |
| 79 | 1.88 | 0.16 | 3.63 |
| 80 | 1.43 | 0.02 | 2.85 |
| 81 | 1.01 | -0.08 | 2.1 |
| 82 | 0.61 | -0.11 | 1.34 |
| 83 | 0.24 | -0.07 | 0.56 |
| 84 | -0.1 | -0.24 | 0.04 |
| 85 | -0.43 | -1.07 | 0.22 |
| 86 | -0.75 | -1.93 | 0.45 |
| 87 | -1.05 | -2.79 | 0.72 |
| 88 | -1.35 | -3.67 | 1.03 |
| 89 | -1.64 | -4.55 | 1.35 |
